# 

**Published:** 1842-10-01

**Authors:** 


### BIBLIOGRAPHICAL RECORD.

1. Pulmonary Consumption: its Prevention and Cure, established on New Views of the Pathology of the Disease. By HEN. GILBERT, M.R.C. Surgeons, London. 8vo. pp. 296. Renshaw, London, June 1842.

2. Animal Chemistry; or, Organic Chemistry in its application to Physiology and Pathology. By JUSTUS LIEBIG, M.D. &c. Edited from the author's M.S. By WILLIAM GREGORY, M.D. &c. of the College Aberdeen. 1 Vol. 8vo. pp. 354. Taylor and Walton, London. 1842.

3. On the Hydropathic Cure of Gout. By G. HUME WEATHERHEAD, M.D. &c. Highley, Fleet-street, pp. 38. Price one shilling.

4. An Introductory Lecture on Pictorial Anatomy, delivered to the Students of the School of Design, &c. By JAMES MILLER, F.R.S. &c. Octavo, pp. 32. Edinburgh, 1842.

5. Annales de la Chirurgie Française et Etrangere. Nos. 18 and 19, for June and July, 1842. Bailliere, Regent Street.

6. Natural History of Man. By Dr. PRICHARD. No. VIII. Price 2s. 6d. Bailliere, 1842.

7. A Retrospect of Practical Medicine and Surgery, being a half-yearly Journal, &c. &c. Edited by W. BRAITHEWAITE, Esq. No. 5, Jan. to July, 1842.

8. An Essay on Diabetes. By H. BELL, D.M.P. one of the Librarians to the Faculty of Medicine, Paris. Translated from the French, by ALFRED MARKWICK, late Externe to the Hôpital des Veneriens, &c. &c. Octavo, pp. 96. Pigott, 1842.

9. Cyclopædia of Anatomy and Physiology. Part XXIV. Sherwood, July, 1842.

10. Cyclopædia of Practical Surgery. By Dr. COSTELLO. Part XI. July, 1842.

11. The Simple Treatment of Disease, deduced from the Methods of Expectancy and Revulsion. By JAMES M. GULLY, M.D. Octavo, pp. 198. Churchill, London, 1842.

12. A Treatise on Amaurosis and Amaurotic Affections. By EDWARD OCTAVIUS HOCKEN, M.D. Highley, 1840.

13. A Treatise on Irritation of the Spinal Nerves, &c. &c. By J. EVANS RIADORE, M.D. Octavo, pp. 306. Churchill, 1842.

14. The Climate of the South of Devon; and its Influence upon Health, with Accounts of Exeter, Torquay, &c. &c. By THOMAS SHAPTER, M.D. Physician to the Exeter Dispensary. 8vo, pp, 258. Churchill, 1842.

15. Medical Report of the Aberdeen Lunatic Asylum for the year ending 30th April, 1841, and also for that ending 1842. By Drs. MACROBIN and JAMIESON.

16. A Practical Treatise on Diseases of the Scalp. By JOHN E. ERICHSEN, M.R.C.S. London, &c. &c. Octavo, pp. 192, with plates. Churchill, 1842.

17. On the Treatment of Hæmorrhagic Diathesis. By JAMES MILLER, Esq.

18. Hastings: considered as a Resort for Invalids. With Tables, &c. By JAMES MACKNESS, M.D. Churchill, 1842.

19. A Case of Carcinomatous Stricture of the Rectum. By ALFRED JUKES, Surgeon to the General Hospital, Birmingham, with Plates. Quarto. Churchill, London, 1842.

20. Commentaries on some Doctrines of a Dangerous Tendency in Medicine. By Sir ALEX. CHRICHTON, M.D. Octavo, Churchill, London, July, 1842.

21. Deformities of the Spine and Chest, &c. By CH. H. ROGERS-HARRISON. Illustrated by Drawings. Churchill, London, 1842.

22. On the Treatment of the Hæmorrhagic Diathesis. By JAMES MILLER, Esq. Read before the Medico-Chirurgical Society of Edinburgh, 1842.

---

23. *Reports of Lunatic Asyla.*
1. Report of the Pennsylvania Hospital.
2. Report of the Retreat for the Insane at Hartford.
3. Report of the Asylum for Persons deprived of their Reason, near Frankford, America.
4. Letter on a Visit to Hospitals, Prisons, &c. in France, England, and Scotland. (Anonymous.)
5. State of the New York Hospital and Bloomsbury Asylum—for 1841.

---

24. On Structure and Life of the Malpighian Bodies of the Kidney. By W. BOWMAN, F.R.S. Assistant Surgeon to King's College Hospital, &c.

---

25. Report of the Managing Committee of the House of Recovery and Fever Hospital, in Cork-street, Dublin, from 1st April 1841, to 31st. March 1842.

---

26. The Curative Influence of the Climate of Pau, and the Mineral Waters of the Pyrennees, on Disease, &c. By A. TAYLOR, M.D. Octavo, pp. 342. Parker, London, 1842.

---

27. Remarks on Amputation: an Essay submitted to the Faculty of Physicians and Surgeons of Glasgow. By ALEX. KING, Esq. 8vo. pp. 44. Glasgow, 1842.

---

28. A treatise on Mineral Waters, with reference to those prepared at the Royal German Spa at Brighton. By Dr. FRANZ. Duodecimo. Churchill.

---

29. A Bedside Manual of Physical Diagnosis. Second Edition, revised and enlarged. By CHARLES COWAN, M.D. Duodecimo, Sherwood and Co. 1842.

☞ *This is a very useful Vade Mecum for the Auscultator.*

---

30. Ninth Annual Report of the State Lunatic Hospital, Worcester. America, 1842.

---

31. Elements of Chemical Analysis, Inorganic and Organic. By EDWARD AND. PARNELL, Chemical Assistant, University College, London. 8vo. pp. 309, with Index. Taylor and Walton, 1842.

32. Methodus Medendi; or the Description and Treatment of the Principal Diseases incident to the Human Frame. By HENRY MC. CORMICK, M.D. Consulting Physician to the Belfast Hospital, &c. 8vo. pp. 574. Longman and Co. 1842.

---

33. The Medical Examiner. Edited by J. B. BIDDLE, M.D. and W. W. GERHARD, M.D. February to July, 1842.

---

34. Forty-Sixth Report of the Friend's Retreat, near York. 1842.

---

35. On the Different Forms of Insanity in Relation to Jurisprudence, &c. &c. By JAMES COWLES PRICHARD, M.D. &c. 8vo. pp. 243. Bailliere, Regent Street, Sept. 1842.

---

36. The Astromagnetic Almanac for 1843, in which all the Motions of the Earth are demonstrated in accordance with the Theory of the Ancient Eastern Nations. By H. H. SHERWOOD, M.D. New York, 1842.

---

37. Atlas illustrative of the Anatomy of the Human Body. By J. CRUVEILHIER, M.D. &c. The figures drawn from Nature by M. Beau, with Explanations by C. Bonamy, M.D. Part 2, Plates vii. viii. lix. lx. Bailliere, London, 1842.

---

38. Report of the Result of the Operation for the Cure of Strabismus in a hundred Patients. By J. B. ESTLIN, F.L.S. Surgeon to the Eye Dispensary, Bristol.

☞ *Out of* 100 *operations* 65 *were "perfect or satisfactory"*—9 *satisfactory, but without late report*—7 *improved, but requiring operation on the other eye*—4 *not improved, but requiring, &c.*—5 *improved, but unfavourable cases*—5 *much improved*—3 *slightly improved*—2 *no improvement.*

---

39. Eighteenth Annual Report of the Visitors of the General Lunatic Asylum of Gloucester, 1841.

---

40. Natural History of Man. By JAMES COWLES PRICHARD, M.D. &c. No. IX. Illustrated with many Coloured Plates. Bailliere, Regent Street, 1842.

---

41. The Microscopic Journal, and Structural Record; a Monthly Periodical, containing the most recent Facts and important Discoveries in Human and Comparative Anatomy, &c. With Illustrative Diagrams, by JOSEPH DINKLE. Edited by DANIEL COOPER, Assistant Surgeon to the Forces, and GEORGE BUSK, Surgeon to the Hospital-ship *Dreadnought*. Van Voorst, Paternoster-row. Price one shilling each number. pp, 32, with plates. No. 19, for September, 1842.

☞ *This interesting Contemporary has hitherto escaped our notice. We shall be happy to exchange Journals with the Editors.*

---

42. Physiological and Pathological Inquiry concerning the Physical Characteristics of the Human Teeth, &c. By CHAPIN A. HARRIS, M.D. Profess. Dentistry, Baltimore.

---

43. The American Journal and Library of Dental Science. Published under the Auspices of the American Society of Dental Surgery. Four numbers, quarterly. New York, 1842.—*In exchange.*

---

44. Elements of Physiology for the use of Students, and with especial reference to the wants of Practitioners. By RUDOLP WAGNER, M.D. Profess. Comp. Anatomy and Physiology, in the University of Gottenburgh. Part 2nd. of Nutrition and Secretion. 8vo. pp. 220. Sherwood, London, 1842.

---

45. A brief Statement of Facts, with regard to certain Researches on the Heart, in Reply to Statements in a Memoir of Dr. Hope. By C. J. B. WILLIAMS, M.D. &c. Churchill, 1842.

---

46. A Series of Anatomical Sketches and Diagrams, with Descriptions and References. By THOMAS WORMALD, Assistant Surgeon, &c. Bartholomew's—and ANDREW MELVILLE MC WHINNIE, Teacher of Anatomy, &c. Part V. Price 4s. Highley, London, September, 1842.

---

47. Ueber die Abhœngigkeit der Physischen Populationskrœfte, Von den einfachsten Grundstoffen der Natur Specler Anwendung auf die Bevolkerungs—Statistik von Belgien. Von Dr. FERDINAND GOBBI. 4to. Leipzig and Paris, 1842.

---

### TO CORRESPONDENTS, &c.

### CHOLERA IN CALCUTTA.

"It sometime in March took quite a new character—those attacked had not the usual purging and vomiting, but it came on with a sort of *faint sinking*, the strength was, without outward effort or apparent cause, totally prostated, and the patient died without suffering, as if labouring under the effect of poison."

The above is from a gentleman of the Civil Service, dated May 10th, and is remarkably characteristic of the pathological views given at page 348, Johnson on Tropical Climates, wherein it is stated that the impression of the morbific cause would appear to be made principally and originally on the organic system of nerves.

J. R. MARTIN.

---

### MIND OR MATTER.

ERRATUM.—By a mistake of the Reviewer, at pages 602-3, the name of ELLIOTSON was substituted for ENGLEDEW, in the Address to the Phrenological Society. The Chairman was Dr. ELLIOTSON, but the Orator was Dr. ENGLEDEW.

